# Redefining familial adenomatous polyposis: competition, cooperation, and the path to monoclonality

**DOI:** 10.1007/s10689-025-00479-3

**Published:** 2025-06-01

**Authors:** Sylvain Ferrandon, Matthew F. Kalady, Sanne M. van Neerven

**Affiliations:** 1 Division of Colon and Rectal Surgery, Department of Surgery, The Ohio State University Wexner Medical Center, Columbus, US; 2 The Gurdon Institute, University of Cambridge, Tennis Court Road, Cambridge, CB2 1QN UK

**Keywords:** Familial adenomatous polyposis (FAP), Tumour initiation, Intestinal adenomas, Polyclonality, Cancer prevention

## Abstract

Familial adenomatous polyposis (FAP) is a hereditary cancer syndrome characterized by germline mutations in the *APC* gene that result in the development of hundreds of premalignant adenomas throughout the colon and rectum. Prophylactic surgery remains the primary intervention strategy, as there are currently no pharmacological treatment options for FAP patients. Previous therapeutic approaches have predominantly focused on reducing polyp size rather than preventing their initiation, thereby missing a key opportunity for early intervention. Crucially, to effectively target the earliest stages of tumour development requires a deeper understanding of the molecular mechanisms underlying adenoma formation. In this review, we evaluate the latest models and methods employed to investigate the origin of FAP adenomas. We describe how mutant cells expand from their initial emergence within the intestinal epithelium and how they compete with normal cells within intestinal crypts. In addition, we discuss how multiple mutant crypts cooperate to collectively form polyclonal adenomas, and how these polyclonal lesions gradually transition towards monoclonality as adenomas progress towards colorectal cancer. Finally, we highlight how these insights inform the development of targeted cancer prevention strategies for individuals with FAP.

## Introduction

Familial adenomatous polyposis (FAP) is a hereditary cancer syndrome caused by germline mutations in the tumour suppressor gene *Adenomatous Polyposis Coli* (*APC*). These mutations predispose individuals to the ongoing accumulation of somatic loss-of-function mutations in the remaining wildtype *APC* allele, resulting in the development of hundreds to thousands of premalignant polyps throughout the colon and rectum over time. If left untreated, the progression to colorectal cancer (CRC) is nearly inevitable, with a lifetime penetrance approaching 100% [1]. Currently, clinical management of FAP patients revolves around prophylactic colectomy or proctocolectomy in combination with life-long endoscopic surveillance to mitigate the risk of malignant transformation [2]. Despite these interventions, the burden of disease remains significant, and there is a pressing need to explore alternative strategies aimed at cancer prevention.

Although extensive efforts have been made to identify novel chemopreventive agents, no such interventions have been successfully translated into routine clinical practice. Remarkably, most prevention approaches focus predominantly on reducing the size and number of polyps once they are formed, rather than addressing the underlying cellular and molecular processes that drive the earliest stages of adenoma development. Understanding the transition from normal intestinal epithelium to premalignant adenomas is crucial for developing interventions that could prevent adenoma formation or inhibit adenoma progression.

Given the limited availability of patient-derived samples, and the need for mechanistic investigations and high-throughput drug screening, there is a critical need for developing advanced laboratory models. These include both in vitro culture systems such as patient-derived organoids (PDO), induced pluripotent stem cells (iPSCs) and embryonic stem cells (ESCs), as well as genetically engineered animal models (GEMMs). PDOs, grown from the intestinal or colonic epithelium of FAP patients retain the genetic and phenotypic traits of the original tissue, including *APC* mutations, making them powerful tools for studying disease progression [3–5]. High-throughput screening using PDOs has resulted in the identification of agents that may inhibit colorectal adenoma growth [6, 7]. However, since these PDOs lack the surrounding microenvironment that crucial for the adenoma-to-adenocarcinoma transition, culture conditions should be optimized to more closely replicate FAP pathophysiology [8, 9].

The emergence of iPSCs offers a non-invasive method for generating colonic organoids by reprogramming of patient-derived cells, mostly from the blood [10–12]. Similarly, ESC lines, generated from embryos following pre-implantation genetic diagnosis of FAP, can be differentiated into colonic organoids enabling the study of the earliest tumourigenic events, including *APC* loss [10, 13, 14]. Importantly, iPSCs and ESCs can be differentiated into multiple cell types, including epithelial, stromal, immune and endothelial cells, allowing for complex co-culture systems that better recapitulate FAP biology [15]. In addition, the use of cancer-on-chip models incorporating vascularization and immune components provide new avenues for studying disease mechanism and therapeutic testing [16–18].

For in vivo studies, the use of GEMMs remains indispensable. Although various animal models have been described to study the role of APC in tumourigenesis, mouse models are the most commonly used. Among these, the *Apc*^Min^ model has been the most widely employed to investigate FAP. This model harbours a heterozygous nonsense mutation at codon 850, referred to as *Min* (multiple intestinal neoplasia), which leads to spontaneous development of 30 or more intestinal polyps by 4–6 months of age [19]. Other spontaneous models carrying mutations at different positions in the *Apc* gene, such as *Apc*^1638N/+^ and *Apc*^∆716^, were later developed and provided a broader spectrum of disease phenotypes [20, 21], highlighting the importance of mutation position within the *Apc* gene. Additionally, conditional or inducible *Apc* knockout models, combined with tissue-specific Cre drivers, allow researchers to study the effects of *Apc* mutations in specific tissues and at different developmental stages [22]. These models are often used in combination with fluorescent markers that enable the tracing of normal or mutant cell populations, or even in parallel [23]. Despite their robustness, a key limitation of these models is that the majority of adenomas arise in the small intestine, whereas in FAP patients, tumours predominantly develop in the colon and rectum. Although the underlying reasons are not fully understood, this limitation can be partially addressed in conditional models using targeted Cre drivers, such as *Cdx2-Cre* [24] and *mCai-Cre* [25] which promote adenoma development specifically in the colon.

The integration of state-of-the-art multi-omics approaches with murine or human FAP models offer a promising opportunity to study the pathophysiology of FAP more detail, thereby undoubtedly providing new insights into the earliest stages of tumourigenesis. In this review, we examine how these experimental models have expanded our understanding of neoplasia in FAP and discuss emerging strategies for cancer prevention that shift the focus from managing established polyps to preventing adenoma formation at its inception.

### The earliest event in colorectal tumourigenesis: cell competition within the crypt

Given the time it takes to develop CRC, it is generally assumed that tumour initiation occurs in long-lived intestinal stem cells (ISCs) or differentiated cells that have reacquired stem-like properties. To study the behaviour of these stem cells, lineage tracing studies using genetically modified mouse models have provided crucial insights into stem cell dynamics within the intestine [26]. These models are based on the labelling of specific cell populations to allow tracking of the progeny of individual cells over time [27]. Such lineage tracing studies have revealed that, under homeostatic conditions, ISCs located at the base of intestinal crypts compete for niche occupancy. This competition is characterized by stochastic loss-and-replacement events, a process reminiscent of neutral drift dynamics [28–31]. Consequently, each intestinal crypt ultimately becomes monoclonal; inhabited by the progeny of a single stem cell. The rate at which this drift to monoclonality occurs is influenced by several factors, including the number of competing ISCs and the rate of replacement [28–31]. Interestingly, analysis of normal-appearing crypts from FAP patients showed a two-fold increase in ISCs replacements, suggesting that loss of the first *APC* allele might convey a selective advantage [32].

**Fig. 1:**
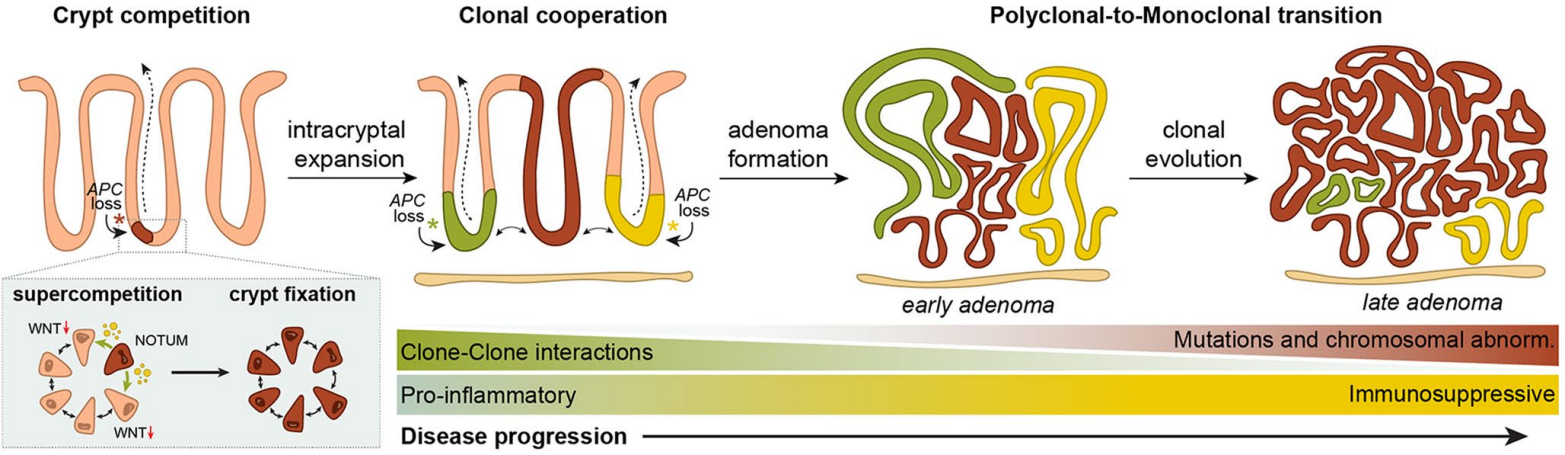
Clonal dynamics during adenoma initiation and progression. Adenoma formation is initiated by loss-of-function mutations in the *Apc* gene within intestinal stem cells (ISCs). These *Apc*-mutant ISCs gain a supercompetitive advantage, allowing them to outcompete neighbouring wild-type ISCs. This occurs through the secretion of Wnt antagonists such as NOTUM, which drive the differentiation of surrounding wildtype cells. As a result, *Apc*-mutant ISCs rapidly expand and become clonally fixed within individual crypts. Over time, neighbouring mutant crypts can interact, giving rise to polyclonal adenomas. During progression, clonal competition and selection eventually lead to the dominance of a single clone, resulting in monoclonal lesions. Early-stage polyclonal lesions are typically associated with a pro-inflammatory microenvironment, which gradually shifts toward an immunosuppressive state as the tumour evolves. This transition may warrant distinct therapeutic strategies for polyclonal versus monoclonal adenomas

Throughout the years it has become evident that oncogenic mutations in genes such as *Apc*, *Kras*, or in other cancer driver genes, can confer a competitive advantage to mutant ISCs [33, 34]. As a result, these mutant ISCs bias drift in their favour, resulting in clonal fixation of the mutation within an intestinal crypt. Crucially, multiple studies have revealed that some oncogenic mutants, among which *Apc*-mutants, display ‘supercompetitor’ behaviour [35–38]. Supercompetitors do not only possess a cell-autonomous advantage, but also actively disadvantage neighbouring wildtype ISCs [39]. In case of *Apc*-mutant ISCs, this is reflected by the secretion of a range of Wnt antagonists, among which NOTUM is the most prominent, that result in differentiation of normal ISCs which rely heavily on Wnt signalling for their maintenance [35, 36]. Consequently, the competitive landscape within individual crypts shifts in favour of the mutant cells, facilitating the development of premalignant adenomas (Fig. 1). Importantly, genetic deletion of *Notum* [35] or pharmacological enhancement of Wnt signalling using lithium [36] can mitigate the competitive advantage of *Apc*-mutant cells and decrease adenoma burden in vivo. Together these works indicate that modulating cell competition within intestinal crypts can provide a novel strategy to prevent polyp development.

### Cooperative interactions and polyclonality drive adenoma formation

Although significant progress has been made in elucidating the mechanisms underlying intra-crypt competition, how intestinal adenomas subsequently arise from these monoclonal mutant crypts remains incompletely understood. A previous study demonstrated that adenoma formation requires more than the presence of isolated *Apc*-mutant crypts, and only fields of mutant crypts transform into premalignant adenomas [40]. Moreover, reducing the size of mutant patches inhibited adenoma development in a non-linear manner, implying that the number of mutant crypts is not merely a probabilistic factor but instead suggests that mutant crypts may act cooperatively to facilitate tumour development [40].

Recent studies have now shed new light on this hypothesis, providing compelling evidence using state-of-the-art molecular methods that clonal cooperation is indeed required for intestinal tumour formation [41–44]. To study this phenomenon, Gaynor and colleagues used two distinct Cre-drivers to label and trace homozygous *Apc*-mutant ISCs at either high clonal density or as isolated mutant crypts. They revealed that, unlike the high-density clones that readily developed adenomas, the isolated mutant crypts almost never transform [41]. This work highlights the necessity for a ‘critical mass’ of Wnt mutant clones to cooperatively facilitate the development of intestinal adenomas.

Further evidence of clonal cooperation has been observed in models where *Apc* alleles are lost consecutively, thereby more closely resembling the pathogenesis of FAP. For example, the study by Sadien and colleagues employed a multi-colour Confetti reporter to trace clonal dynamics after heterozygous *Apc* loss [42]. Subsequent use of mutagenic compound N-ethyl-N-nitrosourea (ENU) led to the development of multiple lesions along the intestinal tract. Analysis of these lesions revealed a large fraction of the fluorescently labelled adenomas containing more than one colour, indicating a polyclonal origin of FAP adenomas [42]. Of these multi-coloured adenomas, around 80% carried more than two *Apc* mutations, and even up to 9 mutations within a single adenoma could be detected. Importantly, microdissection of individually coloured clones within the same adenoma revealed that each clone carried a distinct *Apc* mutation, thereby excluding the possibility of subsequent *Apc* mutation accumulation in a single dominant clone undergoing clonal evolution [42].These findings are supported by two additional studies leveraging advanced single-cell tracing techniques [43, 44]. Lu and colleagues used substitution mutation-aided lineage-tracing (SMALT) based barcoding [43] to uncover cell phylogenies whilst Islam et al. developed Native sgRNA Capture and sequencing (NSC-seq) that allows recording the evolutionary trajectory of individual cells as well as capturing their transcriptomic profiles [44]. Both teams implemented their tracing systems in *Apc*^Min^ mice [19], that carry germline mutations in one *Apc* allele and spontaneously lose function of the second allele, which is the model most closely resembling human FAP. Like the work of Sadien et al., both studies demonstrated that adenomas were initiated by multiple founder clones that frequently exhibited more than two *Apc* mutations. Together, these studies have reported up to a hundred clones within individual adenomas, indicating a high degree of clonal diversity at the earliest stages of polyp development [42–44]. Importantly, this polyclonality was observed in adenoma tissues from patients with both sporadic CRC and FAP [43–45].

Besides the observations regarding clonal composition, all studies reported differences in behaviour between transforming and non-transforming (isolated) crypts [41], as well as between distinct clones within a polyclonal adenoma [42–45]. For example, even though isolated *Apc*-mutant crypts expressed known markers such as Wnt antagonist *Notum* [35, 36], they appeared transcriptionally more like untransformed, wildtype crypts, indicating that the full ‘adenoma phenotype’ is not achieved in single mutant clones [41]. In addition, transforming crypts demonstrated an increased accessibility of thousands of enhancers compared to single mutant crypts, which could explain the observed adenoma-specific gene regulation [41]. Interestingly, organoids cultured from the low-density (non-transforming) model do display this adenoma phenotype in vitro, suggesting a potential role for the local microenvironment in repressing this behaviour in vivo [41].

Within polyclonal adenomas, distinct clones displayed diverse proliferation rates and differentiation status, and both stem cell-enriched and fetal-like clones were reported [42, 44]. In addition, investigation of the ligand-receptor interactions revealed extensive cell-cell communication between distinct clones in polyclonal lesions, mainly involved in regulating cell adhesion and ECM composition [43, 45]. Moreover, large differences in gene expression signatures were observed in polyclonal lesions, especially influencing KRAS and MYC signalling [42, 43]. These observations all point towards dynamic, reciprocal interactions between mutant clones to collectively create ‘just right’ conditions for adenoma initiation.

These studies confirm previous observations of polyclonality in FAP [46–50] and reveal that a significant proportion of sporadic and hereditary intestinal adenomas are polyclonal in origin, thereby emphasizing that clonal cooperation is required during the earliest stages of adenoma formation (Fig. 1) [41–45]. Although the variation across these studies regarding the proportion of adenomas that were classified as monoclonal or polyclonal may reflect differences in experimental methods, it is more likely that this could be attributed to the stage of analysed adenomas. This would support a hypothesis in which adenoma progression is characterized by a gradual transition from polyclonality towards monoclonality, thereby ultimately resembling the monoclonal nature often observed in established CRCs [51].

### Polyclonal-to-monoclonal transition correlates with adenoma progression

Multi-omics analyses of normal tissue, benign and dysplastic adenomas, and adenocarcinomas now provide compelling evidence that indeed points towards a model in which CRC development follows a trajectory of polyclonal to monoclonal transition [43–45, 52]. For instance, monoclonal lesions rarely exhibit more than two *APC* mutations [44], in contrast to polyclonal adenomas that are more genetically diverse [42–44]. In addition, patients harbouring polyclonal lesions were generally younger than patients with monoclonal adenomas, and their polyps had undergone fewer cell divisions, tended to be smaller in size, and often exhibited low-grade dysplasia [43]. On the contrary, monoclonal lesions displayed a higher degree of chromosomal abnormalities such as aneuploidy and copy number alterations and frequently harboured additional mutations in other cancer driver genes [43].

One of the largest differences was detected in the prevalence of *KRAS* mutations, which are significantly more frequent in monoclonal adenomas and comparable to the frequency found in established CRCs [43]. This suggests that *KRAS* confers an evolutionary advantage that overcomes the necessity for polyclonality and results in the selection of a single dominant clone that drives adenomas progression. Experimental evidence supports this notion as loss of *Kras* in combination with ENU-induced mutagenesis resulted in a larger fraction of monoclonal lesions [42]. Moreover, the correlation between the number of cell-cell interactions and the degree of polyclonality further implies that monoclonal lesions are less dependent on inter-clonal signalling [43]. Interestingly, mutations in *SOX9*, *BCL9L* and *CTNNB1* are more commonly observed in early lesions than in advanced CRC, suggesting that some genes that contribute to tumour initiation eventually constrain adenoma progression [43].

In addition, Esplin et al. conducted a comprehensive analysis comparing the abundance of transcripts, proteins, lipids and metabolites between normal and adenomatous tissues from FAP patients [45]. This multi-dimensional approach revealed a plethora of signalling routes that were specifically altered as cells transition from benign to malignant lesions, particularly pathways involved in regulation of cellular homeostasis, metabolism and energy production [45]. Interestingly, these pathways were not consistently altered across all omics methods, emphasizing the importance of analysing regulatory changes across multiple dimensions [45]. Furthermore, large changes in the 3D chromatin architecture have been detected across FAP tissues with variable disease stage. Zhu and colleagues describe how, in normal cells, genes are primed for either activation or repression based on whether they display high or low connectivity between promotors and enhancers (P-E), respectively [52]. Moreover, they reveal how these connections are gradually lost during cancer progression and indicate that especially the highly connected P-E regions result in dysregulated, increased gene expression [52]. These upregulated genes were associated with cell cycle and DNA maintenance pathways [52], comparable to those found in monoclonal polyps [44]. Together, these studies reveal a complex interplay between genetic alterations, chromatin structure, and transcriptional and metabolic reprogramming that collectively drive the transition from polyclonal to monoclonal adenomas.

In addition to competing and cooperating with other epithelial clones, certain oncogenic mutants can further amplify their advantage by engaging in reciprocal interactions with the local microenvironment [37]. Although largely unexplored, it is likely that *APC*-mutant cells participate in similar interactions, leveraging signals from surrounding cells to promote their expansion. Recent research endeavours have particularly focused on the interactions between mutant cells and the immune system, as *APC*-mutant epithelial cells can elicit a plethora of immune responses, and *APC* mutations also influence the behaviour of immune cells. Interestingly, the earliest stages of adenoma development are characterized by a pro-inflammatory immune response, accompanied by an upregulation of the arachidonic acid pathway that mediates prostaglandin production and promotes tissue inflammation. As lesions progress towards a more malignant phenotype, this pro-inflammatory environment shifts towards immunosuppression [43–45]. Co-culture assays using FAP organoids and immune cells have highlighted a critical role for myeloid-derived cells and lymphocytes in regulating this shift, thereby facilitating the transition from adenoma to adenocarcinoma [53–55]. Furthermore, *APC* mutations have been demonstrated to influence T cell functions through several mechanisms: (1) altered cytokine production by regulatory T cells, (2) decreased secretion of cytotoxic granules, resulting in reduced killing efficacy of cytotoxic T cells, (3) a global decrease of T cell infiltration [56, 57]. In line with this, monoclonal adenomas often display signs of T-cell exhaustion [43–45]. These findings indicate that the immune landscape undergoes significant remodelling during polyclonal-to-monoclonal transition and progression towards adenocarcinoma (Fig. 1).

### Towards novel prevention strategies for FAP

With the recent advancements in our understanding of the molecular biology underlying FAP, multiple opportunities for adenoma prevention have emerged that target the earliest stages of tumour initiation. One approach is to focus on modulating cell competition dynamics between normal and *APC*-mutant cells within individual crypts, thereby interfering at the earliest moments of tumour initiation. A pioneering study in this area is investigating the chemoprevention effects of lithium in a small cohort of FAP patients [58]. Lithium, a GSK3-beta inhibitor frequently used for treatment of bipolar disease [59], boosts Wnt signalling in normal cells and renders them less sensitive to Wnt antagonists being secreted by mutant cells. By altering the balance between normal and mutant cells, lithium may help to delay or prevent adenoma formation.

Another potential strategy involves interfering with clonal cooperation during the development of polyclonal adenomas. Since such polyclonal lesions are demonstrated to coincide with a pro-inflammatory microenvironment, the use of non-steroid anti-inflammatory drugs (NSAIDS) such aspirin, celecoxib, and sulindac may be particularly effective in such early-stage lesions but not in monoclonal adenomas that frequently exist in an immunosuppressive state [43, 45]. This distinction could explain the variable results regarding the efficacy of NSAIDs in FAP clinical trials [60]. Supporting this hypothesis, previous research demonstrated that aspirin treatment in *Apc*^Min^ is only effective as a preventive compound if administered continuously from conception, whereas treatment in adult mice failed to yield significant benefits [61]. This finding would suggest that monoclonal lesions most likely are already formed in adult animals. Importantly, a recent study developed a methodology to estimate the timing of initial clonal expansions leading to adenoma development and revealed that these events may occur during early infancy [43]. If validated, this insight could have profound implications for chemoprevention strategies, potentially requiring the need to start treatment in children to prevent adenoma formation.

Furthermore, targeting the transition from polyclonal to monoclonal adenomas could be a promising intervention strategy as well. Based on a recently published study, such approach could involve restoring the connectivity between promotors and enhancers that are frequently disrupted during adenoma progression [52]. This can be achieved with inhibitors of Bromodomain and Extra Terminal domain (BET) family members, which modulate chromatin accessibility and transcriptional regulation, especially in highly connected P-E domains that are associated with malignant progression [52]. Although such approach has potential, it is important to recognise that BET inhibitors influence large transcriptional networks, not just oncogenes. Therefore, long-term toxicity and off target effects must be carefully studied before implementing such strategy in the clinic. Another, perhaps more readily applicable strategy would be to reactivate the immune system in monoclonal lesions that frequently exist within an immunosuppressive environment. While immune checkpoint inhibitors have historically shown limited efficacy in microsatellite stable (MSS) lesions such as *APC*-driven CRC, recent clinical trials suggest this paradigm is shifting. In the UNICORN and NEST phase 2 trials, neoadjuvant combination treatment with botensilimab (anti-CTLA-4 antibody) and balstilimab (anti-PD-1 antibody) demonstrated encouraging pathological responses in MSS colorectal cancer patients, with a fraction of complete response rates of up to 29% and major response rates reaching 47% when the interval to surgery was extended [62, 63]. Importantly, these combinations were well tolerated, with no significant delays in surgery or unresolved immune-mediated adverse events [62, 63]. These findings highlight the growing potential of immune-based approaches, including checkpoint blockade and cancer vaccines that stimulate responses against specific mutant cell populations, as targeted prevention strategies [64]. Together, these emerging approaches offer new possibilities for early intervention.

## Conclusion and future perspectives

Recent advances in experimental models have significantly expanded our understanding of the earliest steps of tumourigenesis in FAP, bridging long-standing gaps in knowledge regarding the polyclonal origin of adenomas and their progression towards monoclonal cancers (Fig. 1). This review reports that multiple mutant crypts act cooperatively to form polyclonal adenomas, and that this is a rate-limiting step in the transition of normal epithelium towards premalignant lesions [41–44]. This challenges the traditional paradigm that adenomas arise solely from single mutant clones undergoing clonal expansion and suggests a more dynamic model where crypts are incorporated into adenomas through cooperative inter-crypt interactions.

A critical implication of these findings is that polyclonality should be considered a fundamental early stage in the adenoma-to-carcinoma sequence, rather than a rare or incidental observation. This appears to be a logical step in adenoma formation in FAP patients, where the continuous accumulation of *APC*-mutant crypts enables cooperative interactions when they arise in close proximity. However, it also challenges our understanding of sporadic adenomas, of which a subset al.so exhibits polyclonality [43, 44]. Since continuous loss of *APC* does not occur in sporadic cases, this suggests that such adenomas rely on other mechanisms to generate sufficient numbers of cooperating mutant crypts. One possible explanation could be that microenvironmental factors, such as microinflammation, contribute to the formation of patches of mutant crypts, as is often observed in the epithelium of patients with inflammatory bowel disease [65–67]. Another possibility is that *APC*-mutant clones recruit adjacent *APC*-wildtype crypts via non-cell-autonomous mechanisms and that these clones act cooperatively to form polyclonal lesions. This hypothesis is supported by genome analyses of individual glands derived from human sporadic adenomas revealing that a large fraction of glands lack mutations in cancer driver genes such as *APC* or *KRAS* [43]. This further emphasizes the necessity to investigate the signals that mediate crypt recruitment and their implications for adenoma expansion.

A striking observation across multiple studies is the transition from polyclonality to monoclonality as adenomas progress toward malignancy [42–45, 52]. This shift is likely driven by the acquisition of additional cancer driver mutations, which induce selective sweeps that facilitate the expansion of a single dominant clone. This transition is often accompanied by profound immune suppression, further facilitating adenoma progression. Understanding the mechanisms governing this transition presents new opportunities for therapeutic intervention, particularly in restoring immune surveillance. Despite these advances, several key questions remain unresolved. While significant progress has been made in understanding epithelial competition and clonal cooperation, how mutant clones interact with their surrounding microenvironment is still largely elusive. Notably, the role of stromal components, extracellular matrix (ECM) remodelling and signalling, and local immune interactions in driving adenoma evolution remains understudied. Spatial transcriptomic approaches, as recently applied to established CRCs [68], may provide a promising avenue for capturing the organization and functional relationships of polyclonal lesions at single-cell resolution. Applying such methodologies to early-stage adenomas of sporadic and hereditary origin could yield critical insights into tumour evolution and microenvironmental interactions.

Importantly, early detection and intervention remain paramount in FAP management. Given the complexity of tumour initiation, preventive strategies must be tailored to target stage-specific vulnerabilities of adenomas, whether by modulating cell competition, preventing clonal cooperation, or restoring immune surveillance. Future research should prioritize uncovering the spatiotemporal and molecular determinants of clonal interactions to ultimately translate these findings into novel preventive and therapeutic strategies for FAP patients.

## Data Availability

No datasets were generated or analysed during the current study.
